# Genome-wide multi-parametric analysis of H2AX or γH2AX distributions during ionizing radiation-induced DNA damage response

**DOI:** 10.1186/1756-8935-6-S1-P58

**Published:** 2013-03-18

**Authors:** Francesco Natale, Alexander Rapp, Wei Yu, Marco Durante, Gisela Taucher-Scholz, Maria C Cardoso

**Affiliations:** 1 Frankfurt Institute for Advanced Studies, Frankfurt am Main, Germany, 60438, Germany; 2 Biophysics Department, GSI Helmholtzzentrum für Schwerionenforschung GmbH, Darmstadt, 64291, Germany; 3 Department of Biology, Technische Universitaet, Darmstadt, Germany, 64287, Germany; 4 Institute for Condensed Matter Physics,Technische Universitaet, Darmstadt, Germany, 64287, Germany

## Background

After induction of DNA double strand breaks (DSBs), the DNA damage response (DDR) is activated. One of the earliest events in DDR is the phosphorylation of serine 139 on the histone variant H2AX (γH2AX) catalyzed by phosphatidylinositol 3-kinases-related kinases. Despite being extensively studied, H2AX distribution[1] across the genome and γH2AX spreading around DSBs sites[2] in the context of different chromatin compaction states or transcription are yet to be fully elucidated.

## Materials and methods

γH2AX was induced in human hepatocellular carcinoma cells (HepG2) by exposure to 10 Gy X-rays (250 kV, 16 mA). Samples were incubated 0.5, 3 or 24 hours post irradiation to investigate early, intermediate and late stages of DDR, respectively. Chromatin immunoprecipitation was performed to select H2AX, H3 and γH2AX-enriched chromatin fractions. Chromatin-associated DNA was then sequenced by Illumina ChIP-Seq platform.

HepG2 gene expression and histone modification (H3K36me3, H3K9me3) ChIP-Seq profiles were retrieved from Gene Expression Omnibus (accession numbers GSE30240 and GSE26386, respectively).

## Results

First, we combined G/C usage, gene content, gene expression or histone modification profiles (H3K36me3, H3K9me3) to define genomic compartments characterized by different chromatin compaction states or transcriptional activity. Next, we investigated H3, H2AX and γH2AX distributions in such defined compartments before and after exposure to ionizing radiation (IR) to study DNA repair kinetics during DDR.

Our sequencing results indicate that H2AX distribution followed H3 occupancy and, thus, the nucleosome pattern. The highest H2AX and H3 enrichment was observed in transcriptionally active compartments (euchromatin) while the lowest was found in low G/C and gene-poor compartments (heterochromatin). Under physiological conditions, the body of highly and moderately transcribed genes was devoid of γH2AX, despite presenting high H2AX levels. γH2AX accumulation was observed in 5’ or 3’ flanking regions, instead. The same genes showed a prompt γH2AX accumulation during the early stage of DDR which then decreased over time as DDR proceeded. Finally, during the late stage of DDR the residual γH2AX signal was entirely retained in heterochromatic compartments. At this stage, euchromatic compartments were completely devoid of γH2AX despite presenting high levels of non-phosphorylated H2AX.

## Conclusions

We show that γH2AX distribution ultimately depends on H2AX occupancy, the latter following H3 occupancy and, thus, nucleosome pattern. Both H2AX and H3 levels were higher in actively transcribed compartments. However, γH2AX levels were remarkably low over the body of actively transcribed genes suggesting that transcription levels antagonize γH2AX spreading. Moreover, repair processes did not take place uniformly across the genome; rather, DNA repair was affected by genomic location and transcriptional activity. We propose that higher H2AX density in euchromaticcompartments results in high relative γH2AXconcentration soon after the activation of DDR, thus favoring the recruitment of the DNA repair machinery to those compartments. When the damage is repaired and γH2AX is removed, its residual fraction is retained in the heterochromatic compartments which are then targeted and repaired at later times.
